# The Medical Student's Guide in Extracting Teeth: With Numerous Cases in the Surgical Branch of Dentistry

**Published:** 1851-04

**Authors:** 


*The Medical Student's Guide in Extracting Teeth: with Numerous Cases in the Surgical Branch of Dentistry.* With Illustrations. By S. S. Hornor, Practical Dentist. 12mo., pp. 76. Philadelphia: Lindsay & Blakiston, 1851.

After a few introductory remarks, the author of the above work commences with a defence of the key instrument, which, after "all others fail," seems to constitute the "*anchor*" of *his* "hopes." That a professed dentist should award so high a meed of praise to an instrument which has been almost wholly laid aside by nearly all good operators, may appear somewhat strange, and at first we were not a little surprised; but after reading a description of the other instruments which he recommends, and the directions laid down in the work for the performance of the operation in question, we do not wonder that he entertains so favorable an opinion of "this much abused" instrument. All the accidents that have occurred with it, he seems to attribute to its injudicious use, and does not, therefore, regard them as constituting any argument against its employment.

With a view of strengthening the position he has assumed in relation to the value of the key, he institutes the following bold comparison: "Because thousands of lives have been lost by the application of steam, through ignorance or negligence of the engineer, and the imperfections of the machinery, yet I am not convinced of the policy of its abandonment." Now, if a safer and equally efficient motive power could be obtained, and at no greater cost, we apprehend that our author would be convinced of the wisdom and propriety of abandoning the use of steam. In the extraction of teeth, we have instruments, in our opinion, that may be used with more efficacy and greater certainty of success, and which, as the author admits, "cause less pain than the key." Why, then, should we resort to "this much abused instrument?" We believe that no good reason can be assigned for doing it, and those who have attempted it, have only done so by assuming, in the face of well attested facts, and the most ample and enlightened experience, the converse of the proposition. If it were necessary, we could cite fifty instances where teeth have been extracted with forceps, after unsuccessful attempts to remove them with the key had been made, and by individuals who are regarded as skillful in the use of the last named instrument.

For the extraction of the upper central incisors, the author recommends the use of straight forceps, and after applying them, directs the force to be outward and inward, and afterwards perpendicular, using the precaution, to prevent the instrument from slipping, of not applying the force too suddenly. By combining with these movements a rotary motion, the removal of the tooth will be considerably facilitated. But when the crowns of these teeth are very much weakened, or the roots only remain, "the key," says the author, "may generally be used to advantage," using a very narrow claw, with a short round fulcrum. In some cases, where the root has become hollow and thin from decay or drilling, the instruments which he mentions for their removal are the key, the screw, the elevator, the punch and the hook ; but in a case of this sort, the conical screw, or what is vastly preferable, Dr. Hullihen's compound screw forceps, is the best instrument that can be employed. With this instrument the root may always be extracted at once, provided the decomposed portions are first removed with a suitably shaped instrument. We do not regard the key, elevator, punch or hook, as being at all adapted to the removal of the root of an upper incisor.

The directions for the removal of the other teeth, though perhaps not quite as objectionable, are, in our estimation, more or less faulty and imperfect. But on the operation, altogether, only about sixteen pages of the work are devoted.

The author next treats very briefly on the occurrence of excessive hemorrhage after the extraction of teeth ; after which the "symptoms indicating the necessity" for the operation, are noticed. These include many of the morbid conditions to which the teeth are liable, and many of the effects to which they give rise. In this part of the work, a number of highly interesting cases are introduced. But in the course of his observations on the indications for the operation, the author says : "If the tooth is broken in the attempt to extract it, the operator should never allow his patient to suffer until the nerve suppurates, but should surely destroy it by means of the preparation before mentioned," (arsenious acid, sulph.

morph. and kreosote,) "which will prevent the pain which is generally the consequence of a lacerated nerve, and arrest the serious effects which might otherwise be produced by leaving a root in the jaw." Instead of all this painful, temporising treatment, the proper method of procedure, in a case of this sort, would be, the immediate removal of the remaining portions of the tooth.

